# Active training and driving-specific feedback improve older drivers' visual search prior to lane changes

**DOI:** 10.1186/1471-2318-12-5

**Published:** 2012-03-02

**Authors:** Martin Lavallière, Martin Simoneau, Mathieu Tremblay, Denis Laurendeau, Normand Teasdale

**Affiliations:** 1 Division de kinésiologie, GRAME, Département de Médecine Sociale et Préventive, Faculté de Médecine, Université Laval, 2300 rue de la Terrasse, Québec, Québec, G1V 0A6, Canada; 2 Département de Génie électrique et génie informatique, Faculté des sciences et de génie, Université Laval, Québec, Québec, Canada; 3 Vieillissement, Centre de recherche FRSQ du CHA universitaire de Québec, Québec, Canada

## Abstract

**Background:**

Driving retraining classes may offer an opportunity to attenuate some effects of aging that may alter driving skills. Unfortunately, there is evidence that classroom programs (driving refresher courses) do not improve the driving performance of older drivers. The aim of the current study was to evaluate if simulator training sessions with video-based feedback can modify visual search behaviors of older drivers while changing lanes in urban driving.

**Methods:**

In order to evaluate the effectiveness of the video-based feedback training, 10 older drivers who received a driving refresher course and feedback about their driving performance were tested with an on-road standardized evaluation before and after participating to a simulator training program (Feedback group). Their results were compared to a Control group (12 older drivers) who received the same refresher course and in-simulator active practice as the Feedback group without receiving driving-specific feedback.

**Results:**

After attending the training program, the Control group showed no increase in the frequency of the visual inspection of three regions of interests (rear view and left side mirrors, and blind spot). In contrast, for the Feedback group, combining active training and driving-specific feedbacks increased the frequency of blind spot inspection by 100% (32.3 to 64.9% of verification before changing lanes).

**Conclusions:**

These results suggest that simulator training combined with driving-specific feedbacks helped older drivers to improve their visual inspection strategies, and that in-simulator training transferred positively to on-road driving. In order to be effective, it is claimed that driving programs should include active practice sessions with driving-specific feedbacks. Simulators offer a unique environment for developing such programs adapted to older drivers' needs.

## Background

More than ever, road safety is a public health concern. One cause for this concern arises from changes in demographics. It is expected that the number of older drivers will increase substantially in the next decades. Specifically, it is estimated that this number will double within the next 25 years from 27 million to nearly 60 million in the United States [1]. With ageing, sensorimotor and cognitive changes are known to reduce driving performance [2]. A host of changes in the visual system occurs with ageing [3]. Moreover, some authors suggest that older drivers reduce their visual search patterns which results in a perceptual narrowing (or tunnel effect) [4]. An in-vehicle study conducted by Bao and Boyle [5] showed that compared to younger drivers (18-25 and 35-55 years old), the road scanning of older drivers (65-80 years old) at intersections were primarily confined to areas located directly in front of or slightly to the right or left of the vehicle's direction of motion. Similarly, Romoser and Fisher [6] examined if older drivers made a secondary look as often as younger drivers at the onset of the turn at an intersection. Their results revealed that, while turning, older drivers took less often that second look than younger drivers for potential hazards (6.9% vs. 22.2%, respectively).

Other studies have assessed visual search strategies while changing lanes but did not consider the age effect [7-9]. For instance, Kiefer and Hankey [10] evaluated two groups of drivers (40-50 and 60-70 years old). They reported visual inspections towards the blind spot for only 32 and 15% for the left and right lane changes, respectively. Unfortunately, no specific mention of blind spot inspection made by their older drivers is mentioned. Therefore, there is little information on how older drivers verify blind spot while changing lanes even though it is mentioned that it is a problematic and recurrent issue with older drivers during on-road evaluation [11,12]. In a simulator experiment, Lavallière et al. [13] showed that older drivers inspected their blind spot less frequently than younger drivers while changing lanes (41% vs. 86%, respectively). An important question that remains is whether or not older drivers will show similar frequency of visual inspections towards the blind spot on the road and if training could help improving older drivers' visual search strategies in such situations.

Several retraining programs adapted to older drivers have been developed and are now proposed to this category of drivers. These programs are mostly classroom oriented (refresher program) and aim at promoting safe driving as well as increasing older drivers' confidence behind the wheel through a curriculum emphasizing awareness of traffic hazards, insisting on the need for anticipating the actions of other drivers and providing a general overview of traffic regulations. There are suggestions, however, that these refresher programs do not reduce crash occurrences [14] and do not modify older drivers behaviors. In a cohort of 884 older drivers (55 to 94 years old) who attended a classroom program, Nasvadi and Vavrik [15] found no significant decrease in crash rates in any age group. This might not come as a surprise because motor learning occurs as a direct result of active practice and concrete feedback on the motor performance. As suggested by many authors, physical practice is the preferred form of practice for optimizing learning [16]. Accordingly, several aspects of driving may not be optimized in conventional classroom oriented programs as learning general driving information does not result in sufficient modification to sensorimotor driving strategies. If inadequate visual search precedes a driving error, corrective feedback for this specific action and practice are needed if a decrease of such errors is to be achieved. The development of an effective and specific error-detection process likely translates into sensorimotor strategies related to driving. This key concept is often defined as transfer-appropriate practice [17].

In a recent study, Bédard et al. [18] measured knowledge of safe driving practices and driving performance before and after a training program (intervention group) combining a refresher course and on-road training (two 30- to 40-minute on-road practice sessions with a certified instructor). Compared to a control group (no intervention), participants in the intervention group improved their driving knowledge (measured through the 55ALIVE Driver Safety Program questionnaire) as well as some aspects of on-road driving (starting/stopping/backing and moving in the roadway). This study, however, does not allow determining the selective effects of the refresher course and on-road training on the driving improvement. Romoser and Fisher [6] compared the effectiveness of active, passive and no training on older drivers' performance in intersections. Active training increased a driver's probability of looking for a hazard during a turn by nearly 100% in both post-training simulator and on-road driving sessions. Customized feedback was successful in altering drivers' perception of their abilities. Those receiving passive training (i.e., refresher course only) or no training did not change their visual inspection strategies at intersections. It was found that active training (feedback and practice) was more effective than passive training for increasing older drivers' likelihood of looking for threats before a turn.

In the present study, we evaluated if simulator training, coupled with video-based feedback can modify visual search behaviors of older drivers while changing lanes (Feedback drivers). The results were compared to those of participants in a control group who attended a refresher course and drove the same simulator scenario as the Feedback group without receiving feedback about their driving performance (Control drivers). The effectiveness of the training was assessed by comparing on-road driving performance before (Pre-training) and after (Post-training) the training program using a standardized evaluation procedure to document visual search patterns during lane changes. Based on the motor learning arguments presented above, we hypothesized that, compared to Control drivers, Feedback drivers should increase the frequency of visual inspections towards the blind spot while changing lanes after attending the training program.

## Methods

### Participants

22 older drivers (aged between 65 and 85 years old) were recruited through local senior's organizations. They were all healthy and active drivers (additional details are provided in the Results section and Table 1). Upon their arrivals in the laboratory, participants were briefed on the requirements of the experiment and invited to read and sign an informed consent declaration conformed to the Institutional Review Board. The IRB gave approval for the study to take place (CERUL 2005-070 phase 2, 03-07-2006). They were assigned randomly to a Feedback (6 men and 4 women) or a Control group (9 men and 3 women). They received 20$ per session for their participation.

**Table 1:** General driving experience and performance on screening test

Mean values (standard deviations)	Control drivers	Feedback drivers	P values
Demographic			

Age	69.3 (4.5)	72.1 (5.3)	0.198

Gender (Female/Male)	3/9	4/6	

Years of experience	47.3 (7.5)	47.8 (5.2)	0.407

Kilometers per week (km)	211.3 (147.3)	166.8 (108.6)	0.453

Performance on screening tests			

Mini Mental State Examination, MMSE	28.2 (1.1)	28.1 (0.9)	0.879

Snellen visual acuity High contrast	0.92 (0.22)	1.10 (0.31)	0.126

Melbourne Edge Test	21.0 (1.34)	20.9 (1.66)	0.881

Motor-Free Visual Perception Test, standard score	103.6 (22.5)	118.9 (20.9)	0.125

Motor-Free Visual Perception Test, time (seconds)	3.5 (1.7)	3.7 (1.4)	0.815

Head rotation towards the left*	43.2 (3.7)	48.5 (3.7)	0.857

Head rotation towards the right*	47.8 (4.5)	56.1 (4.5)	0.559

* The only significant effect was observed for side of head rotation; rotation to the right being greater than to the left for both groups.

### General assessment

On the first session, all participants were given a general verbal questionnaire that included items on driving (years of driving experience, frequency of driving and average km/week, and presence of an accident within the last years). This information regarding self reports of driving exposure was used to verify if participants were active drivers and to control for potential low mileage bias [19,20]. A number of clinical tests were conducted to verify that drivers followed driver's license regulations as well as to verify that both groups were similar with respect to their general health condition (neurological and musculoskeletal problems, use of medication). Usual clinical tests for cognition (Mini Mental State Examination (MMSE)[21]) and static visual acuity (Snellen visual acuity, Melbourne Edge Test [22], Motor-Free Visual Perception Test (MVPT)) were also used to screen for cognitive or visual impairments that might affect driving skills. Each of the subject standard vision and health conditions were in accordance to the local legislation. Finally, voluntary range of motion of the head was measured with an electromagnetic sensor (Ascension Technology Corporation, Flock of Birds, Burlington, Vermont, USA) fixed on a head band. Subjects were seated in the simulator and were asked to move their head as far as possible to the right and to the left. The maximal head rotation on each side was measured.

### Procedure

**Figure 1:**
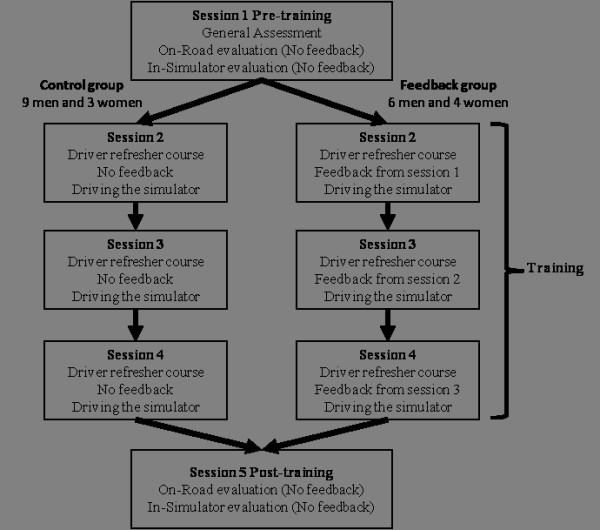
**Experimental timeline**.

Subjects participated in five sessions on five different days within a 14-day period. Figure 1 presents a general overview of the experimental timeline. The first (pre-training) and last session (post-training) included on-road and in-simulator evaluations without any feedback. Three training sessions with a general driver refresher course (both groups), exposure to driving in a simulator (both groups) and driving specific feedbacks (Feedback group only) were given in between the pre-and post-training sessions. Description of the instrumented car, simulator and procedures for the on-road evaluations and training follow.

### Instrumented car

The on-road evaluations took place in a car instrumented with a global positioning system (GPS, Novatel, Calgary, Alberta, Canada), four digital cameras (1 for the driver's head and 3 for the driving environment: forward view and right and left blind spot, PointGrey Research, Richmond, British Columbia, Canada) and one 3D accelerometer (Crossbow CXL04LP3, Crossbow Technology Inc., Milpitas, California, USA). Synchronized videos were recorded at 25 Hz on a desktop computer powered by an external battery. All other data were collected synchronously with an A/D board on a portable computer (sampling frequency of 20 Hz for the GPS and 500 Hz for the accelerometer). Positioning of the cameras did not interfere with the vision of the driver.

### Driving simulator

A fixed-based open-cab simulator powered by STISIM Drive 2.0 (System Technology Inc., Hawthorne, California, USA) was used for training purposes. Images were projected on a flat wall (1.45 m high × 2.0 m wide) located 2.2 m from the steering wheel using a projector (Hitachi CP-X275) displaying a 40° horizontal by 30° vertical field-of-view with the center of the screen located at eye-level through the midline of the subject. Three video cameras (Prosilica CV-640, Burnaby, British Columbia, Canada) were mounted on the cab facing the subject and zoomed to fully capture head and eye movements. A fourth camera (Point Grey Research, Richmond, British Columbia, Canada) captured the scenario displayed on the screen. A magnetic tracker (Flock of Birds, Ascension Technology Corporation, Burlington, Vermont, USA) secured on driver's head allowed to record head movements when driving. To simulate real-driving conditions, the left-side mirror and a panel positioned in the left blind spot were instrumented with two white light emitting diodes (LED). The LEDs informed the driver about the traffic condition and the possibility of changing lanes. When the LEDs were on, the driver was instructed to delay his maneuver until LEDs were turned off. The information displayed by the LEDs was in correspondence with the information displayed in the rear view mirror embedded into the simulator's scenario. This information was provided in order to train participants to gaze at these regions and to process the information before changing lanes. The experimenter made sure the participants understood the meaning of the blind spot when driving. This was also part of the driver's refresher course (see training procedures below). When a driver changed lanes while the LEDs were still on, a crash would occur and be recorded in the simulator file. No crash occurred during this experiment.

### On-road evaluation

All drivers drove the same vehicle and were tested on the same open road circuit (12 km) for both the pre- and post-training sessions. No feedback was given during these sessions. The circuit consisted of urban driving with pre-determined directions during non-rush-hour traffic. It included a complete range of driving maneuvers. Each on-road evaluation included ten lane changes (8 towards the right and 2 towards the left).

A qualified driving instructor first offered general instructions and orientations to the driver. Thereafter, the instructor sat on the passenger seat and provided, ahead of time, verbal indication about the upcoming maneuvers. This evaluation lasted approximately 30 minutes.

### Simulator driving and training

After the on-road evaluation on the first session, participants were invited to drive in the simulator. Firstly, all participants were familiarized with the driving simulator with a scenario including less graphical information and fewer maneuvers than for the upcoming main scenario (practice drive). A 5-min break was given before they drove the main scenario. This main scenario included recorded instructions to inform the driver about the specific maneuvers to be performed (e.g., instructions to overtake securely a slower moving vehicle). Subjects were asked to comply with local traffic regulations throughout the scenario. No emergency braking response was necessary unless a driving error was made. This scenario lasted approximately 25 min. Participants were informed about simulator sickness and were specifically instructed to inform the experimenter when this happened. They were told the experiment would stop immediately without any prejudice for them. To prevent simulator sickness prone situations to occur, the temperature within the room was maintained around 19°C with proper air conditioning using a ceiling vent positioned just above the driver. For each simulator session, a visual analog scale (VAS) was completed by the driver at baseline (i.e., before driving), after the practice drive and after the experimental run (VAS 10-cm scale; 0 = no symptom, 10 = mild nausea). A score above 5 after the practice run led to a specific query about the interest and capacity to continue. A score of 10 terminated the participation. A 5-min rest period between the practice run and the experimental run was provided. In the present study, only 2 older subjects reported being uncomfortable after the practice run. These individuals reported a score of 10 on the VAS (mild nausea) and were therefore excluded from the experiment. Although these 2 subjects from the Control group did not drive in the simulator for the training sessions, they nevertheless attended the refresher course and the pre- and post-training on-road sessions. No feedback was given in this first simulator session and it only served to expose subjects to the simulator and to provide simulator materials for the first training session (Feedback group only).

For session 2 to 4, all participants first received an individual driving refresher course offered by the same instructor. The course was based on the AARP's 55ALIVE Driver Safety Program and lasted about 40 minutes (each session). The course included specific sections with graphical support to inform participants about traffic regulations, mirror and blind spot verification when changing lanes and vehicle control. After each course, participants were invited to drive in the simulator. Feedback drivers received feedback about their previous on-road (on session 2 only) and simulator driving (on session 2, 3 and 4) prior to driving in the simulator. Control drivers drove the same scenario but did not receive any feedback prior to the end of the study.

**Figure 2:**
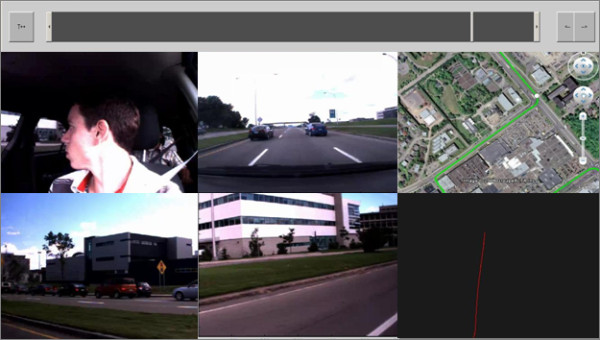
**Visual layout of the software tool used by the instructor for providing feedback to participants**. The example depicted here involves a young driver looking at the right blind spot during an on-road session: Upper-section: Navigation bar, Middle-Left: Video of eye and head movements, Middle-Middle: Front view of the vehicle, Middle-Right: GPS signal overlaid on a map, Lower-Left: View of the left blind spot, Lower-Middle: View of the right blind spot, and Lower-Right: Car's speed in km/h.

For the Feedback group, the driving-specific feedback emphasized the role of preventive rather than reactive driving with the intention to increase mirror and blind spot inspections prior to changing lanes. The information provided was inspired from the Risk Awareness and Perception Training Program [23-26]. To provide driving-specific feedback, a custom software module allowed video information and vehicle data to be displayed synchronously (e.g., position and speed as well as a display of the position of the vehicle on a map) with fast forward, playback, and zooming functions. Figure 2 depicts an example of the display presented to drivers. It has been observed that older drivers adopt a perceptual narrowing strategy while driving [4,13,27,28]. To specifically work on this sub-optimal strategy, a specific feature implemented in the software allowed highlighting the importance of visual inspections (with or without head movement) in order to improve visual search strategies. As an example, instructions were given on the importance of checking the blind spot prior to changing lanes. When inappropriate strategies were observed, the researcher demonstrated the proper response and used the software for displaying the driver's own at-risk response (eye/head movements, scenario and vehicle kinematics). Thereafter, he invited the participant to drive over the particular sections of the scenario where the errors occurred. These additional feedback sessions specific to the Feedback group added about 15 minutes to each training session. These sessions (driving-specific feedbacks) were offered to participants in the Control group after completion of the study (after session 5).

### Data analyses

Voluntary range of motion of the neck. Data for the range of motion were submitted to a Group (Control, Feedback) by Side (Left, Right) analysis of variance (ANOVA) with repeated measures on the last factor.

Frequency of inspections. For each on-road lane change, 20 seconds of data were extracted from the records; 15 seconds prior to the initial displacement of the vehicle towards the target lane and 5 seconds after this initial displacement [13]. In prior experiments, it was observed that inspection of the mirrors and blind spots occurred as early as 15 sec before the onset of the lateral movement of the car [10,13]. Video streams of the head and the forward view from the vehicle were observed simultaneously frame by frame to document visual inspections towards five specific regions of interest (ROI: 1) forward view, 2) odometer, 3) rearview mirror, 4) external mirrors and 5) blind spots). Inspections of the 5 ROI required distinctive eye-head responses. Straight ahead gaze with little or no head movements were classified as inspections of the forward view; downward eye and head movements were classified as inspections of the odometer; oblique and upward eye and head movements were classified as inspections of the rearview mirror; left or right and downward eye and head movements were classified as inspections of the external mirrors; finally, blind spot checking was characterized by large eye and head movements towards the left or right.

For each driver, the mean frequency of visual inspection to the rearview and external mirrors and the blind spot was calculated. If a visual inspection was made toward these ROI during the 20 sec interval, a value of one (1) was assigned to the ROI and (0) otherwise. Frequency was then calculated for each driver from the sum of the inspections divided by the number of lane changes. This measure corresponds to the probability of occurrence of an inspection to a given ROI and is an indication of the use of mirrors and blind spot during lane changes [9]. Since we are mainly interested in how individuals evaluated their driving environment while changing lanes, the forward view and odometer ROI were not considered. To determine if driving-specific feedback and active practice could alter the visual search strategy, frequency data were submitted to a Group (Control, Feedback) by Visit (Pre-training, Post-training) by ROI (Rearview mirror, External mirror, Blind spot) analysis of variance (ANOVA) with repeated measures on the last two factors. Late verifications of the blind spot have been reported by Lavallière et al. [13] in an earlier simulator study. A delayed verification to the blind spot, after the onset of the maneuver, is an ineffective visual inspection since it puts the drivers in a reactive rather than in a preventive mode. In order to determine when the information was extracted, the time stamp of each inspection of the blind spot with respect to the initial displacement of the vehicle was noted. For each group, frequency of verification of the blind spot was computed for 5-s temporal bins (15 seconds prior to the onset of the maneuver to 5 seconds after the onset of the maneuver). Positive values indicate that a visual inspection followed the onset of the lane change whereas negative values indicate that the visual inspection preceded the onset of the lane change.

## Results

All participants completed the program in less than two weeks (on average, 9.5 and 11.4 days for the Control and Feedback groups, respectively). A t-test showed that this difference was not significant (p > 0.05). No difference was found between the two groups concerning items on driving and general health conditions. All older drivers scored 27 or higher on the MMSE [21] and reported similar distance driven in the previous year (i.e., approximately 100 km per week). The voluntary range of head rotation was similar in both groups (F(1,18) = 1.22, p > 0.05). The interaction of Group by Side was not significant (F(1, 18) = 0.84 p > 0.05) and, for both groups the maximal amplitude of rotation to the right was greater than to the left (on average, 52.3 vs. 45.5 degrees, respectively) (F(1, 18) = 16.68, p < 0.001). Moreover, none of the drivers reported difficulty to turn their head and torso or reported neck or torso pain that would have restricted head movements. Table 1 provides a summary of these results.

### Frequency of visual inspections during lane changes

Overall, 144 lane changes were analyzed for the Control group (80 and 64 events for the Pre- and the Post-training sessions, respectively) and 156 lane changes for the Feedback group (74 and 82 events for the Pre- and the Post-training sessions, respectively). Ninety-six (96) lane changes are missing due to either road constructions blocking the road or subjects taking more than one lane when turning at a previous intersection (72 and 24 events for Control and Feedback groups, respectively).

**Figure 3:**
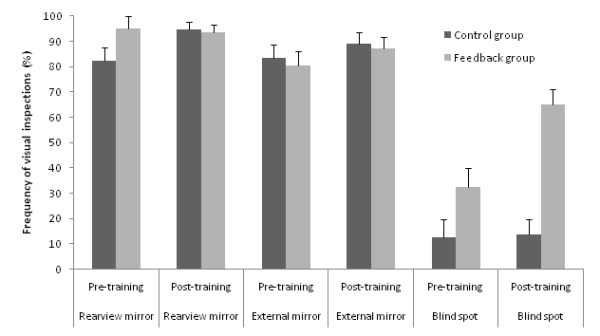
**Frequency of visual inspections (%) to rearview and external mirrors and the blind spot**. Error bars indicate the standard error of the means.

Figure 3 presents for both groups the frequency of visual inspection to the rearview and side mirrors and the blind spot when changing lanes during Pre- and Post-training sessions. The ANOVA yielded significant main effects of Group (F(1,19) = 9.41, p < 0.01), Visit (F(1,19) = 17.05, p < 0.001) and ROI (F(2,38) = 165.17, p < 0.001), a Group by ROI interaction (F(2,38) = 14.75, p < 0.001) and a Group by Visit by ROI interaction (F(2,38) = 4.88, p < 0.05). The interaction of Group by Visit and Visit by ROI were not significant (p > 0.05). The decomposition of the interaction of Group by Visit by ROI indicated that in Pre-training, both groups verified less frequently their blind spot than the other ROI. The analyses of the frequencies of inspection of the rear view and external mirrors revealed neither a group difference nor a training effect (ps > 0.05). On average, both groups inspected their rear view and external mirrors for 91% and 85% of the lane changes, respectively. Training was effective, however, in changing the frequency of verification of the blind spot for drivers in the Feedback group only. While drivers in the Control group showed no change in blind spot verification (12.5% vs. 13.8% of verification for the Pre- and Post-training sessions, respectively), drivers in the Feedback group increased the verification of their blind spot (32.3% vs. 64.9%, for the Pre- and Post-training sessions, respectively).

### Temporal inspection of the blind spot

**Figure 4:**
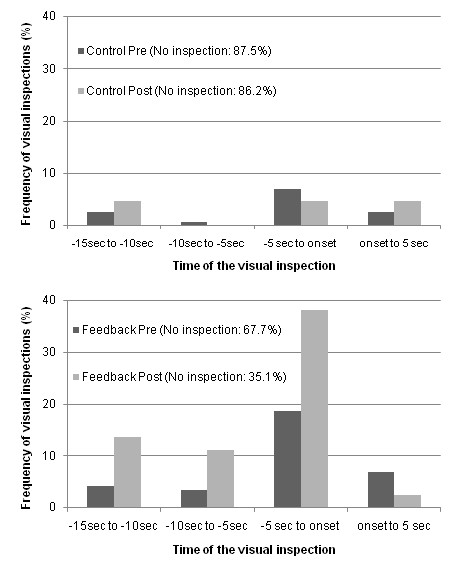
**Frequency distribution (%) of the temporal occurrence of the first inspection toward the blind spot**. The top panel is for the Control group and the bottom panel for the Feedback group.

Temporal distribution of the first inspection towards the blind spot is illustrated in Figure 4. The abscissa presents 5-s temporal bins with respect to the onset of lane changes. The ordinate represents the percentage of inspection for all lane changes. For each group, the percentages of lane changes without a verification of the blind spot are presented in the legend of the figure. Drivers in the Control group (top panel) did not modify the temporal location of their visual inspection. It is important to bear in mind that these drivers seldom verified their blind spot for both visits (i.e., less than 14% of the lane changes). For drivers in the Feedback group (bottom panel), however, in addition to the large increase in the frequency of visual inspections towards the blind spot from the Pre- to the Post-training session (32.6% increase), one can observe a clear increase of visual inspections occurring prior to the onset of the lane changes (after the training, 96% of the verifications occurred prior to the onset of the lane changes).

## Discussion

The aim of this study was to determine whether or not a training program based on simulator training combined with driving-specific feedbacks would improve older drivers driving skills. The analysis of visual search strategies during lane changes revealed that the driving-specific Feedback group increased drastically the frequency of blind spot verifications (from 32.3% to 64.9% of the lane changes) whereas the Control group did not.

This result confirms recent observations by Marottoli et al. [29] suggesting that a refresher session only is not effective for improving driving skills. In their study, improvement was seen only when the refresher course was combined with on-road training (two 60 minutes with an instructor). Bédard et al. [18] reported similar results. Unfortunately, in this latter study the absence of a group following the classroom program only does not allow to single out the effect of the classroom program or of the on-road training.

The present findings demonstrate that in-simulator training combined with driving-specific feedback could be an effective substitute to on-road training. Moreover, even if one-on-one driving refresher course were given to participants by the instructor, our results provide additional support to the suggestion that this type of training method fails to alter visual search strategies. Our observations corroborate recent result from Romoser and Fisher [6]. In their study, drivers in a control group (no training) and drivers receiving verbal training only on driving safety (passive training) did not improve their driving skills for turning at intersections. On the other hand, older drivers undergoing active driving in the simulator with specific feedback increased the number of secondary looks at the onset of the turn at intersections. It is worth mentioning that the current results support these previous findings in suggesting that driving-specific feedback and active practice in simulator can transfer positively to on-road driving conditions.

At the Pre-training evaluation, although we randomly assigned subjects to each group, there was a significant group difference for the frequency of blind spot verifications during lane changes (32.3% vs. 12.5% for Feedback and Control, respectively). Nonetheless, the Feedback group, despite showing a higher frequency of verification before training increased drastically the frequency of verification of the blind spots after the training program. It is also worth mentioning that this action started prior to the onset of the lane change. No change was observed for participants in the Control group. It is worth noting that blind spot verification was specifically reviewed in the refresher program and the importance of this visual search action prior to changing lanes was emphasized with specific graphical support. The low frequency of blind spot verifications obtained from the Pre-training on-road evaluation is similar to previously recorded data on visual searches during lane changes for younger drivers (16% to 31%)[9,10] and older drivers in a simulator study (41%)[13]. Specific feedback on driving performance and active practice helped the participants to modify their visual searches during lane changes. Moreover, this modification in the visual search strategy was not achieved at the expense of reducing inspections to other regions of interest (i.e., rearview or external mirrors). The percentage of visual inspections towards the rearview (82.2% and 94.9%) and external mirrors (83.3% and 80.6%) were higher than for blind spot checking for both groups (Control and Feedback groups, respectively). These higher frequencies might explain the absence of a training effect for these 2 ROI (ceiling effect). However, we did find that the Feedback group inspected the rear view mirror earlier at the Post-training than at the Pre-training session. No difference was observed for the external mirrors. Participants in the Control group showed no change for the time of inspection for the rear view and external mirrors. This earlier gaze to the rear view mirror might reveal a change in the visual search strategy of the Feedback group. By extracting the visual information of their surroundings earlier (earlier look at the rear view mirror) and by increasing the frequency of visual inspection towards the blind spot, these drivers put themselves in a preventive rather than in a reactive state. To our knowledge, this is the first time the visual search behavior of older drivers is evaluated during lane changes in urban on-road conditions.

In their study, Romoser and Fisher [6] stated that they were not able to determine whether it was the feedback, the active practice (access to the simulator) or both factors that led to the improvement in performance of the active training group. By providing the same active practice session in the simulator to the Control group, we had control over these factors. Consequently, we can conclude that the specific feedback combined with active practice improved the visual search strategies of the Feedback group. Access to a driving refresher curriculum about road safety tips and in-simulator practice sessions (without feedback) did not allow drivers in the Control group to modify their visual search strategy. This suggests that these drivers considered themselves as good drivers and were somehow reluctant to the refresher program or failed to translate the information that was provided to their own driving experience. Because visual search strategies like the ones requested for executing a lane change are mainly described as a top-down sequence of information processing steps [30], one needs to have a proper mental model of the sensorimotor sequences that must be executed. If the driver's belief is that the blind spot region does not represent a possible source of hazard prior to executing a lane change, there is little to no chance that this driver will glance at this region [31], even after being taught in a classroom that the blind spot is a critical region of interest for their safety. Previous studies suggest that older drivers are particularly inefficient at assessing their own performance [32-34]. Although they are aware of what hazardous situations may consist of, they seem unable to realize that they could be involved in such situations. Such an attitude (i.e., high self-rating even in the presence of declining skills) is an obstacle to self-modification of driving habits since an essential aspect of learning consists in being able to evaluate one's errors [35]. By receiving specific video based feedback about their own driving performance, the older drivers from the Feedback group increased their ability to identify at-risk driving strategies. Moreover, by participating in active practice sessions in the simulator, they were able to engage the necessary processes leading to appropriate driving behaviors when tested on-road (i.e., increased frequency of blind spot verifications and earlier verifications). For the moment, we are not able to determine how long the improvement in visual search strategy observed for the Feedback group would last and whether refresher sessions are needed. Future experiments should allow us to provide an answer to this question.

## Conclusions

In conclusion, our results suggest that drivers, when being shown their own sub-optimal strategy and receiving specific feedback to change it, engaged the necessary cognitive processes to improve their visual search strategy when changing lanes. As reported by Romoser and Fisher [6], simulator training may offer a secure mode of training where drivers can physically practice driving strategies that mimicked those used on-road. Providing classroom-only information (refresher course) or simply driving in a simulator without specific information about the performance does not appear to be sufficient for drivers to fully appreciate the nature and importance of their driving errors. This suggests that simulator training combined with tools providing driving specific-feedback could be an important means for modifying driving behaviors and reinforcing proper driving strategies. While on-road training is still considered as the gold standard, driving simulators combined with appropriate feedback could offer an efficient, cost-effective and safe means of retraining older drivers and other at-risk groups of drivers (e. g., traumatic brain injured drivers). A recent case report demonstrated that in-simulator training with specific feedback improved the driving skills of a traumatic brain injured driver [36]. Finally, this type of training protocol might be useful for driving instructors by providing them with a tool supporting specific and concrete feedbacks to their students.

## Competing interests

The authors declare that they have no competing interests.

## Authors' contributions

ML, NT, DL and MS participated in the design of the study. ML and MT conducted the acquisition of data and data mining. ML and NT performed the statistical analysis. All authors have been involved in the interpretation of data, drafting (ML and NT) or revising (MS, MT, DL) the manuscript critically for important intellectual content. All authors read and approved the final manuscript.

## Pre-publication history

The pre-publication history for this paper can be accessed here:

http://www.biomedcentral.com/1471-2318/12/5/prepub
